# Controlled mud-crack patterning and self-organized cracking of polydimethylsiloxane elastomer surfaces

**DOI:** 10.1038/srep14787

**Published:** 2015-10-06

**Authors:** Rian Seghir, Steve Arscott

**Affiliations:** 1 Institut d’Electronique, de Microélectronique et de Nanotechnologie (IEMN), CNRS UMR8520, The University of Lille, Cité Scientifique, Avenue Poincaré, 59652 Villeneuve d’Ascq, France

## Abstract

Exploiting pattern formation – such as that observed in nature – in the context of micro/nanotechnology could have great benefits if coupled with the traditional top-down lithographic approach. Here, we demonstrate an original and simple method to produce unique, localized and controllable self-organised patterns on elastomeric films. A thin, brittle silica-like crust is formed on the surface of polydimethylsiloxane (PDMS) using oxygen plasma. This crust is subsequently cracked via the deposition of a thin metal film – having residual tensile stress. The density of the mud-crack patterns depends on the plasma dose and on the metal thickness. The mud-crack patterning can be controlled depending on the thickness and shape of the metallization – ultimately leading to regularly spaced cracks and/or metal mesa structures. Such patterning of the cracks indicates a level of self-organization in the structuring and layout of the features – arrived at simply by imposing metallization boundaries in proximity to each other, separated by a distance of the order of the critical dimension of the pattern size apparent in the large surface mud-crack patterns.

It is well known that cracking of materials can lead to pattern formation^1^. Such cracking can happen over a wide range of length scales – from the macroscopic, e.g. geological^2^,^3^,^4^ and biological^5^ systems, to the microscopic, e.g. cracking of thin films in technology^6^,^7^,^8^,^9^,^10^,^11^,^12^,^13^,^14^,^15^,^16^,^17^,^18^,^19^,^20^,^21^,^22^,^23^,^24^. In the latter case, uncontrolled cracking is usually an unwanted phenomenon^25^ – often resulting in the abandonment of the technological process. However, it is thought that harnessing and controlling cracking could be of great use in the area of technology^13^,^26^,^27^,^28^,^29^,^30^,^31^,^32^,^33^. The reason for this is that micro and nanotechnologies^34^ currently rely heavily on pattern formation using lithographic methods – i.e. the so-called ‘top down’ approach to fabrication^35^. Although a very powerful approach, it is becoming apparent that such techniques will ultimately have limits in terms of complexity^36^. In contrast, cracks can be generated spontaneously, forming highly complex patterns in non-equilibrium^37^,^38^ – albeit still with lower resolution that current top-down approaches^28^. In the context of thin films, instabilities can occur due to the residual film stress being either compressive^39^ or tensile^1^ – the former results in surface wrinkling^40^,^41^, the latter can result in surface cracking^42^. Such phenomena, when controlled, would be of great use in applications where top-down fabrication approaches are not always compatible such as *inter alia* soft-lithography^43^, artificial skin^43^, micro^44^ and nanofluidics^45^, and micro and nanomanufacturing^30^,^46^. It could, in addition, provide key solutions for tomorrow’s applications such as bio-inspired technologies^32^, where the cracks could act as natural stress amplifiers and sensors, synthetic fingerprinting^47^, and cryptography^48^, where it could play the role of an automatic inviolable code generation system, and flexible electronics^49^ where the creation of specific and desired defects could keep weak system parts far from damage^50^.

The objective of the current study is to demonstrate a new approach to generate and tune crack-based patterns without using lithographic intervention. This is achieved using residual tensile stresses in an evaporated thin metal film deposited onto a flexible elastomer substrate having a brittle crust. Here, organized patterning using cracking is achieved in the following way: (i) polydimethylsiloxane (PDMS) is exposed to oxygen plasma to form a brittle silica-like layer on the surface (ii) a chromium film – having residual tensile stress^51^,^52^,^53^ – is evaporated onto this layer (iii) above a certain critical chromium thickness (2–5 nm) the residual stresses of the combined layer are sufficient to cause cracking of the layers to form mud-crack patterns (iv) an additional evaporated gold layer (100 nm), slightly in tension^54^, enhances the crack width and facilitates pattern observation, and (v) if metallization boundaries are imposed the cracking is parallel and quasi-periodic.

## Results and Discussion

### Concept of metallization-induced cracking of PDMS

**Figure 1:**
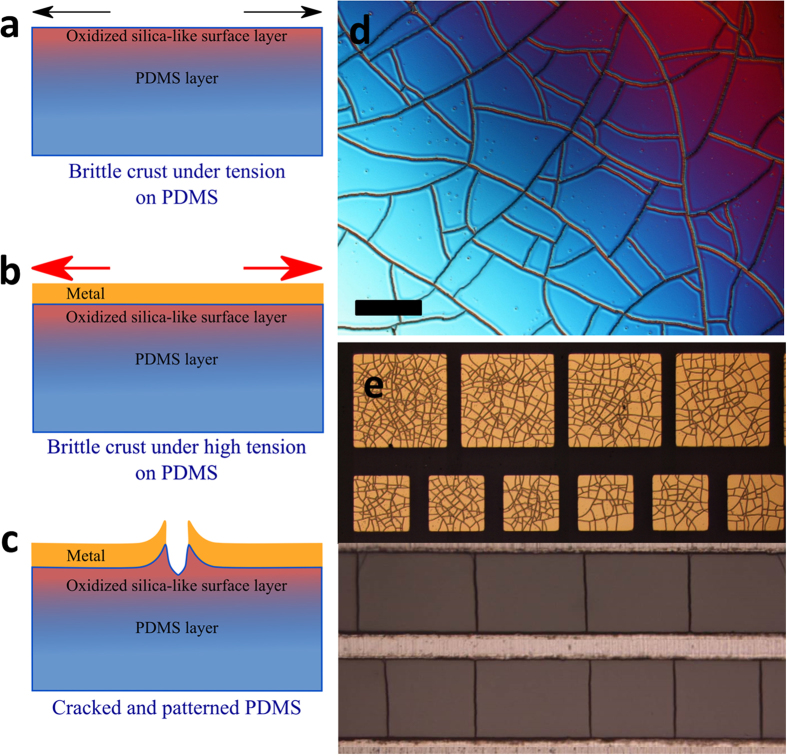
Metallization-induced cracking of the silica-like crust formed on polydimethylsiloxane (PDMS) elastomer exposed to oxygen plasma. (**a**) uniform silica-like crust – having residual tensile stress – is formed on the PDMS via exposure to oxygen plasma dose. (**b**) a thin metal film (chromium/gold) – having residual tensile stress – is evaporated onto the surface of the silica-like crust. (**c**) cracking of the silica-like crust and the metal film occurs if the residual tensile stresses are greater than the ultimate tensile strengths of layers. (**d**) mud-crack patterning is the result of this process for large surface metallization (scale bar = 100 μm) and (**e**) the proximity of metallization boundaries leads to the appearance of self-organized cracking (the large squares are 1 mm^2^ and the lines have a thickness of 150 μm).

Figure 1 shows the basic idea proposed here. Firstly, a uniform, non-cracked silica-like crust – having a residual tensile stress – is formed on the surface of a PDMS sample via exposure to oxygen plasma – Fig. 1a. The basic steps of the oxygen plasma are given in Supplementary Fig. 1 of the Supplementary Information. The effect of an oxygen plasma is to create a nanometre thick^10^,^19^,^55^ silica-like ‘crust’^7^ on the surface of the elastomer. Depending on the plasma dose and oxygen pressure, this crust can be mechanically stressed leading to the formation of organized wrinkles^56^,^57^ (compressive stress) or cracks^7^,^8^,^10^,^19^,^23^,^58^,^59^,^60^ (tensile stress) on the surface of the PDMS. A thin metal film (chromium – possibly with additional gold layer) – having residual tensile stress mainly due to metal melting points, substrate temperature and deposition rate – is subsequently evaporated onto the surface of the silica-like layer (Fig. 1b). The effect of the residual tensile stress in the metal film is to produce a non-equilibrium which results in pattern formation^37^ via cracking^61^ of the bi-layer chromium/silica-like PDMS – Fig. 1c. Indeed, it is well known that the deposition of thin metal films onto the surface of PDMS can result in cracking of the metal^56^,^62^ and, indeed, cracking of the PDMS surface^63^, as can thermal cycling^64^. Figure 1d shows the mud-crack patterning of the PDMS surface as a result of the process presented here for large surface metallization – as we will see, the mud-crack pattern density can be controlled via the plasma dose and the metallization thickness. Metallization-induced cracking enables the formation of low size dispersion and more controllable mesa structures as opposed to a spontaneously cracked surface via high dose plasma exposure (see Supplementary Fig. 3 in the Supplementary Information). Figure 1e shows the effect of the proximity of the metallization boundaries which, as we shall see, leads to the appearance of self-organized cracking and self-defined metal mesa features.

PDMS samples – having a thickness of 1 mm and a surface of 1 cm^2^ – were prepared using a commercial elastomer kit (see Methods). All PDMS samples used in the study had the same base/curing agent ratio (10:1) and all samples were cured using the same thermal procedure (see Methods). The PDMS samples were exposed to oxygen plasma over a large range of doses *D* (360 J to 180 kJ) in a commercial oxygen plasma chamber (see Methods). All oxygen plasma treatments in the study were performed at an oxygen pressure of 0.4 mbar i.e. 40 Pa.

### Plasma induced crack spacing

As attempted by some researchers in different contexts^55^,^60^,^65^, initial experiments were performed on the PDMS in order to determine the boundary between a plasma dose producing a uniform, non-cracked silica-like crust on the PDMS surface and a plasma dose which provokes spontaneous cracking of the silica-like crust on the PDMS surfaces using oxygen plasma (see Supplementary Fig. 3 in the Supplementary Information). The experiments were able to show that this threshold value of *D* was in the range 1.5–1.8 kJ, i.e. spontaneous cracking of the PDMS surface is not observed for an oxygen plasma dose of ≤1.5 kJ (50W/30s) at a pressure of 40 Pa.

**Figure 2:**
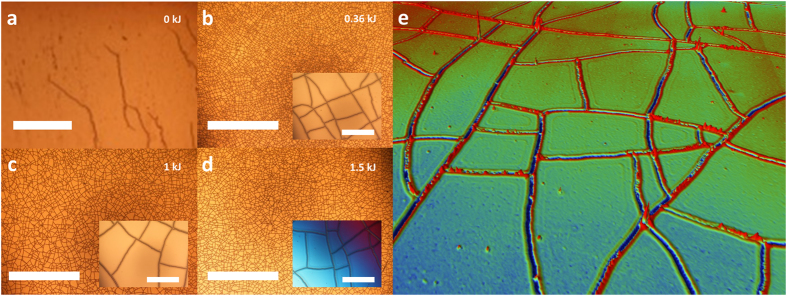
Optical microscope images of oxygen plasma treated PDMS samples following a chromium/gold (10 nm/100 nm) layer has been evaporated onto the surface. (**a**) no plasma treatment (scale bar = 500 μm), (**b**) Dose *D* = 360 J, the crack density *N* = 2.5 ± 0.3 × 10^8^ m^−2^. (**c**) *D* = 1 kJ, *N* = 1.4 ± 0.2 × 10^8^ m^−2^. (**d**) *D* = 1.5 kJ, *N* = 1.2 ± 0.2 × 10^8^ m^−2^ (scales bars = 2000 μm) and (**e**) a 3D optical profile image of the mud-crack patterning following removal of the chromium/gold thin film. The insets to a-d show zoomed images of the cracks (Scale bars = 100 μm). The chromium/gold was evaporated over the whole 1 cm^2^ surface of the PDMS.

Let us first investigating the influence of a low PDMS plasma dose exposure (*D* < 1.5 kJ) and a constant chromium/gold (10 nm/100 nm) bi-layer evaporation, on mud-crack patterns. Figure 2 shows the cracking created via a blanket metallization of PDMS surfaces which had previously been exposed to varying oxygen plasma doses between 360 J (Fig. 2b) and 1.5 kJ (Fig. 2d), including 1 kJ (Fig. 2c). In each case in Fig. 2, the evaporated thin metal layer was chromium/gold (10 nm/100 nm). Figure 2a shows a metallized PDMS surface which was not exposed to oxygen plasma. One can observe micro-cracks, as well as a dense network of nano-cracks (not visible here) in the chromium/gold layer which have been reported^66^. If we now consider the metallized samples where the PDMS had been exposed to oxygen plasma – a distinctive mud-crack patterning was observed for all samples over the oxygen plasma dose range studied – see Fig. 2b–d. Figure 2e shows a 3D optical profile of the cracked PDMS surface following removal of the chromium/gold thin film. The mud-crack patterning is composed of surface cracks having a similar profile to the spontaneously formed cracking of PDMS surfaces at high plasma dose (see Supplementary Fig. 3 in the Supplementary Information) surrounding well-defined, non-cracked polygonal mesa features. Indeed, it is interesting to note that such mesa features, surrounded by a crack network, are free of nano-cracks and perfectly smooth as opposed to metallization of PDMS not exposed to oxygen plasma (see Fig. 2a). The mud-crack patterns observed for the metallized, plasma-exposed PDMS samples strongly resemble those observed in nature^2^,^3^,^67^,^68^,^69^,^70^,^71^,^72^,^73^,^74^.

Note that uncontrolled mud-cracking of technological thin films is relatively common^14^,^15^,^16^,^17^,^21^,^22^,^23^,^62^. Such features have been reported in a number of thin film coatings, e.g. on chromate conversion coatings (CCCs)^14^, gallium nitride films^15^, diamond-like carbon^16^, yttrium stabilized zirconia films^17^,^21^, nanostructured titanium dioxide films^18^, amorphous silicon^20^, lanthanum strontium cobalt iron oxide (LSCF) films^22^ and also on metallized PDMS^23^,^62^ – although none of these studies speak of *controlled* mud-crack patterning.

The mud-crack patterning observed here is apparently more ‘organized’ than the cracks which form spontaneously on PDMS samples exposed to high plasma dose (>1.8 kJ) – see Supplementary Fig. 3 in the Supplementary Information. The polygonal mesa density – or simply ‘crack density’ *N* per surface is 2.5 ± 0.3 × 10^8^ m^−2^, 1.4 ± 0.2 × 10^8^ m^−2^ and 1.2 ± 0.2 × 10^8^ m^−2^ for a dose of 360 J, 1 kJ and 1.5 kJ. Note that the standard deviations are somewhat lower than for those obtained for the spontaneously cracked PDMS samples indicating a higher ordering in the metallization-induced cracking of the PDMS compared to the high plasma dose-induced cracking of the PDMS (see Section 1 of the Supplementary Information). The values of *N* enable a characteristic dimension *L*_*c*_ of the polygonal mesas 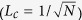 to be determined to be 62.8 ± 3.6 μm, 83.4 ± 5.1 μm and 92 ± 6.6 μm for 360 J, 1 kJ and 1.5 kJ plasma dose respectively. As the value of *N* is based on statistical data (see Methods), *L*_*c*_ can be loosely interpreted as the average ‘diameter’ of a polygonal mud-crack mesa – i.e. there is, on average, no cracking inside surfaces 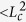. We partially conclude here that when the plasma dose is multiplied by ~4 the average crack-free mesa surface, 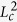, is multiplied by ~2. This demonstrates a slight but clear connection between plasma-dependent silica-like layer properties (thickness, hardness and residual tensile stress level) and mud-crack characteristics.

**Figure 3:**
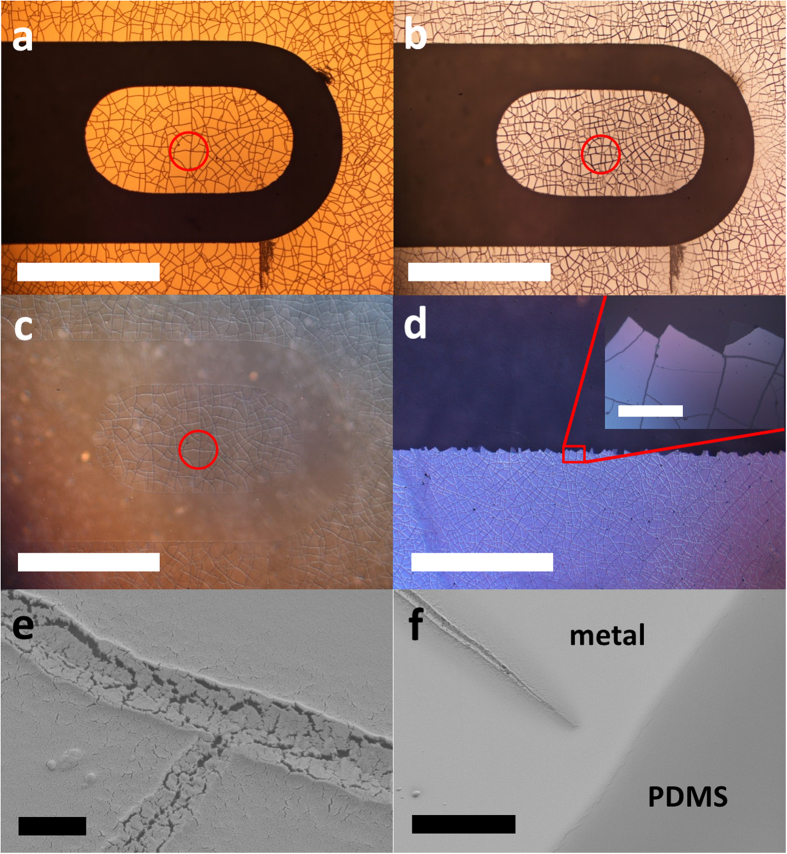
Effect of etching away the chromium/gold metallization. (**a**) a chromium/gold (10 nm/100 nm) coated PDMS sample (*D* = 1 kJ) before etching. (**b**) after gold etch showing chromium layer, (**c**), after chromium etch showing cracks in PDMS surface. (**d**) the effect of surface wetting on a dip etch (scale bars = 2000 μm) and (**e**) Scanning electron microscopy (SEM) of the cracks: zoom image showing a crack junction – micro-cracks are visible in the metallization covering the mud-crack pattern cracks (scale bar = 2 μm) (**f**) SEM image of the cracking in the vicinity of the metallization boundary – cracks end perpendicularly to this boundary (scale bar = 20 μm). The inset to (**d**) shows a zoom of the metallic islands formed via the wetting behaviour (scale bar = 100 μm).

Etching away the chromium/gold layer (see Methods) revealed that the same mud-crack patterns which are present in the metal layer are present in the PDMS surface. Figure 3 shows the effect of selectively etching away the metal layers from the surface of the PDMS. Figure 3a shows a portion of the metallized PDMS surface which was partly masked – via the effect of a clip to hold the PDMS sample during evaporation of the chromium/gold thin film. It is important to note that no cracking is apparent in the masked area – indicating that is it the effect of the thin metal film which instigates the observed cracking of the PDMS surface. Removal of the 100 nm thick gold layer using a wet etch (see Methods) reveals the 10 nm thick chromium layer – see Fig. 3b.

Note that during gold layer removal in a liquid environment, the thin sample was subjected to bending and torsion leading to the creation of a new set of cracks – ‘extra cracks’. These extra cracks are clearly visible on chromium layer in Fig. 3b (see red circle) and still slightly visible on PDMS surface after chromium removal (wet etching – see Methods) in Fig. 3c (see red circle). Indeed, focusing on the resulting patterning of the PDMS surface (see Fig. 3c), it is interesting to notice first, that both ‘evaporation cracks’ and ‘extra cracks’ do not lead to the same apparent PDMS crack width and depth (optical contrast), secondly that subsequent bending and torsion does not lead to extra cracks in non-metallized regions. This confirms first that such a plasma-exposed PDMS (*D* = 1.5 kJ) is not brittle enough to fracture even when it is submitted to relatively high manual tensions and torsions. This confirms that is it the metallic layer’s fracture which leads to the silica-like crust fracture, by imposing high strain concentrations on its surface, contrary to high plasma dose (>1.8 kJ) where the silica-like crust cracks spontaneously during its chemical surface transformation – see Section 1 of the Supplementary information. This allows us to suggest that the stresses leading to metal layer fracture during thermal evaporation are significantly higher than the stress subsequently applied manually.

Subsequent temperature cycling – as high as 250 °C – of the samples revealed that no further cracking was observed on PDMS surface. From a technological and application point of view, such a result demonstrates that, once the crack pattern is created and the metal is removed, the PDMS can be reasonably stretched without affecting its crack network – such results are not guarantee regardless of the oxygen plasma exposure^64^. Another feature which becomes more apparent when the thin metal film is removed is a crack feature which runs parallel along the mask edge – see Fig. 3c, this is seen in the SEM images – Fig. 3f. This point is interesting in the context of nano- and/or micromachining, since we demonstrate firstly that a specific region can be patterned, regardless of the neighbouring ones, and secondly that the contour of the masked region itself is well defined. This point will be discussed and exploited with regards to the controlled characteristic dimension *L*_*c*_ of the mesas structures.

In addition, an interesting feature of the wet etching of the thin metal films – associated with the complex wetting behaviour of surfaces having defects^75^ – is shown in Fig. 3d. When a dip etch is used to etch away part of the thin metal films, the contact line of the liquid follows the cracking – due to contact angle hysteresis^75^ – resulting in a metallized/non-metallized boundary shown in Fig. 3d – the inset to Fig. 3d shows a zoom revealing metallized and non-metallized polygonal mesa features along the wetted and non-wetted boundary of the dip etch. This result could be of technological use to form features, e.g. metallic networks of mesa structures, without resorting to lithography – the wetting contact angles of the PDMS samples exposed to oxygen plasma can be found in Section 4 of the Supplementary Information.

Scanning electron microscopy (SEM) of the metallized PDMS samples – see Fig. 3e and Fig. 3f – revealed some important points concerning the metallization-induced cracking. Firstly, the gold layer covers crack-free and cracked PDMS areas and secondly metallized gold surface contains sub-micrometre micro-cracking within the cracked PDMS areas – etching away the gold reveals that the chromium films did not contain these micro-cracks. This indicates that the cracking of the silica-like crust occurs *during* the metallization of the chromium layer (i.e. before gold deposition) and continues to evolve during the gold layer deposition (additional tensile stress) leading to such locally highly stretched and cracked gold surfaces.

We observe that a single film of chromium, having sufficient residual tensile stress, cracks the silica-like layer to enable a certain crack density *N*. In addition, the experiments indicate that when gold (100 nm) is evaporated onto the chromium thin film, the additional residual stresses in the thicker gold film result in an increase of the crack width without affecting the crack density. When the combined chromium/gold evaporation is removed, the cracks are well more visible on the PDMS surface and the pattern is maintained. As a consequence, and even if no precise investigation of the crack width has been done yet, we suggest that the chromium layer and the gold one play two different technological roles: The first layer, if its thickness is sufficient and depending of the silica-like crust brittleness (see Fig. 2), imposes a crack pattern while the second layer only contributes to increasing the crack width. We will see further that such a crack width increase cannot be achieved only by using a thicker chromium layer without affecting the mesa surface 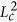 - two different metallized layers are required to control both the mesa surface and the crack width.

Another important observation is that the cracks terminate near to the edge of the masking feature and are always perpendicular to any straight metallization edges – see Fig. 3f. Firstly, the crack network is consequently entirely contained within the shadow masking region which technologically leads to the building of a confined and closed nano-channel network on PDMS surface. Secondly, the natural crack orientation closed to the boundaries, opens the way to a control of crack patterns by adjusting boundary conditions, i.e. the mask shape and dimensions without complex process^31^. However, it should be noted that in the current study the minimum shadow mask size imposes a limit on the technology and observations. To investigate and exploit smaller features would require a different masking approach e.g. photolithography or electron beam lithography. However, such processes could modify the plasma treated surface as various wet processes, e.g. lithographic development, are required and in addition such processes may introduces extra mechanical stresses/strains due to the spin coating/drying of the photo-sensitive polymeric layer.

### Chromium-induced crack spacing

Let us now consider the influence of the thickness of the chromium thin film on the cracking behaviour. In order to do this, all PDMS samples tested were exposed to oxygen plasma at a dose of 1.5 kJ – i.e. approaching the limit of spontaneous cracking behaviour observed without the necessity of metallization. The plasma exposed PDMS samples were subsequently metalized using an evaporated chromium/gold layer having a thickness of 2 nm/100 nm, 5 nm/100 nm, 10 nm/100 nm and 100 nm – the final sample was uniquely chromium.

**Figure 4:**
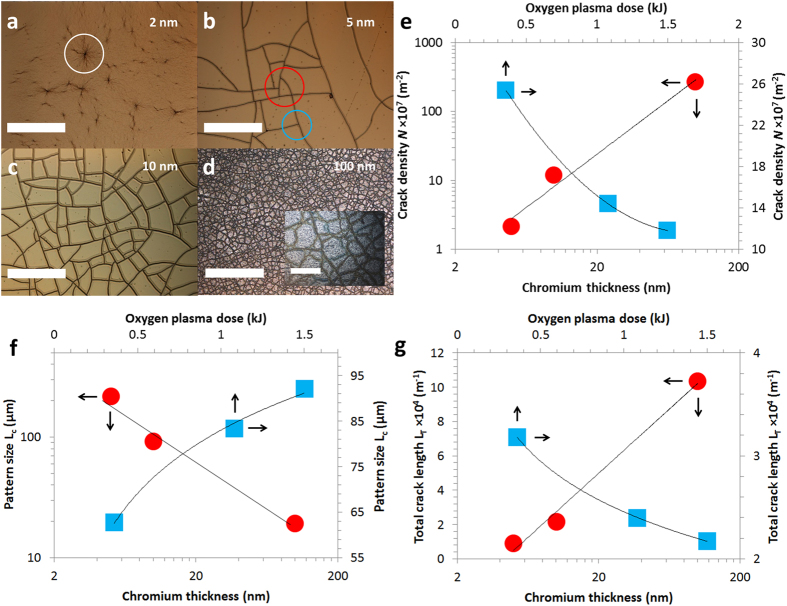
Optical microscope images of oxygen plasma treated PDMS samples as a function of chromium/gold layer thickness. (**a**) chromium/gold = 2 nm/100 nm. (**b**) chromium/gold = 5 nm/100 nm, the crack density *N* = 2.1 ± 0.3 × 10^7^ m^−2^. (**c**) chromium/gold = 10 nm/100 nm, *N* = 1.3 ± 0.2 × 10^8^ m^−2^. (**d**) chromium = 100 nm, *N* = 2.7 ± 0.5 × 10^9^ m^−2^ (Scale bars = 300 μm). (**e**) plots of the mud-crack pattern mesa density *N* (log) versus oxygen plasma dose *D* and evaporated chromium thickness (log). (**f**) plots of the pattern size *L*_*c*_ (log) versus oxygen plasma dose and evaporated chromium thickness (log). (**g**) total crack length *L*_*T*_ (per square meter) versus oxygen plasma dose and evaporated chromium thickness (log). The inset to (**d**) shows a zoom of the mud-crack patterning (scale bar = 30 μm). The red circles correspond to an oxygen plasma dose of 1.5 kJ – the blue square correspond to a Cr metallization thickness of 10 nm.

Figure 4 shows photographs of the metallized, oxygen plasma exposed PDMS samples taken using an optical microscope. Mud-crack patterning is apparent in samples where the chromium thickness is greater than 5 nm – Fig. 4b–d. The topography of the 2 nm chromium sample indicates features which are possibly crack initiation sites – see white circle in Fig. 4a – which can be understood by analogy to fold nucleation of PDMS surfaces exposed to plasma^76^. Indeed, it is interesting to compare the process of fold nucleation, and growth towards a network of closed domains using compressive stresses^76^ (increasing plasma dose) with the process observed here: crack nucleation (Fig. 4a) towards a network of mesa structures bounded by cracks (Fig. 4b–d) using tensile stresses (increasing chromium thickness). Notice that 2 nm thick chromium layer reveals wrinkle features^56^ between these sites – increasing the thickness of the chromium beyond some value between 2–5 nm causes mud-cracking of the surface resulting in flat, wrinkle-free island structures between the cracks. It is important to note however that for thin chromium films, the metal-insulator percolation transition is around 2 nm (see Section 7 of the Supplementary Information) thus the observations for this specific thickness could be due to film non-uniformity.

If we now consider the thicker films, Fig. 4b–d indicate that the crack density appears to be strongly dependent on the chromium thickness. For a fixed plasma dose (1.5 kJ) at an oxygen plasma pressure of 40 Pa, the crack density *N* per surface is evaluated to be 2.1 ± 0.3 × 10^7^ m^−2^, 1.3 ± 0.2 × 10^8^ m^−2^ and 2.7 ± 0.5 × 10^9^ m^−2^ for an evaporated chromium thickness of 5 nm, 10 nm and 100 nm. Note again the relatively low values of standard deviations in the values of *N* indicating a high level of ordering in the induced patterning. Two features are apparent in Fig. 4b,c that are predicted by numerical modelling^77^. Firstly, some smaller mesas are apparent (red circle) and, secondly, there are many 90° approaches of cracks (T-junctions) – blue circle. We can again evaluate the characteristic length *L*_*c*_ 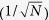 for the mud-crack patterns, the values of *L*_*c*_ are 217.2 ± 13 μm, 92 ± 6.6 μm and 19.3 ± 1.6 μm for chromium thickness of 5 nm, 10 nm and 100 nm. The values of *N* and *L*_*c*_ are plotted against plasma dose and chromium metallization thickness in Fig. 4e and Fig. 4f. It is apparent that over the range of chromium thicknesses (×20 – from 5 nm to 100 nm) and plasma doses (×4 – from 360 J to 1.5 kJ) tested, that the chromium thickness has a larger impact on the mud-crack pattern mesa density than the plasma dose – especially in the range 5–10 nm. Indeed, over the ranges considered and by comparing the average effect of chromium thickness and oxygen plasma dose on the average crack-free surface 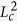, one observes that the sensitivity to plasma dose is about 0.5 while the sensitivity to chromium thickness is about 6. In terms of applications, this is advantageous as the metallization thickness can be controlled very carefully from sub-nanometre to several micrometres. The plot of characteristic mesa length *L*_*c*_ versus chromium thickness *t*_*Cr*_ (see Fig. 4f) reveals the following relationship: 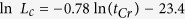 with a coefficient of determination *R*^*2*^ equal to 0.98.

By making some assumptions we are also able to estimate the total length of the cracking per square metre *L*_*T*_. In a first approximation we can consider the polygonal mesa features to be squares of approximately the same surface. If there are *N* squares (per square metre) than the number of sides *s* (per square metre) is given by 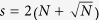 and the total crack length (per square metre) 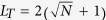. This formula allows us to estimate *L*_*T*_ to be 9.2 × 10^3^ m^−1^, 2.2 × 10^4^ m^−1^ and 1 × 10^5^ m^−1^ for chromium thicknesses of 5 nm, 10 nm and 100 nm. Plots of *L*_*T*_ versus metallization thickness and plasma dose are shown in Fig. 4g. Increasing the thickness of the chromium from 5 nm to 100 nm (×20) leads to an increase in the total crack length *L*_*T*_ of 10 times.

### Masking-induced crack spacing

The experiments so far indicate the following: (i) for a given plasma dose and chromium film thickness there is a polygonal mesa characteristic length *L*_*c*_ for the mud-crack patterns. (ii) the mud-crack patterning is only present on the portion of PDMS surface which was exposed to the metallization and, finally, (iii) the metallization-induced cracking in PDMS surfaces is always perpendicular to the metallization boundaries in the vicinity of the boundary. Based upon this knowledge, let us now consider the possibility of self-organized cracking of the PDMS surfaces by reducing the lateral size of the metallization towards the polygonal mesa feature size for a given plasma dose and metallization thickness. In order to do this, square, rectangular and line shaped metallized (chromium/gold) features (having surfaces ranging from 2 × 2 mm^2^ down to 100 × 100 μm^2^) were evaporated on top of the oxygen plasma exposed (1.5 kJ) PDMS samples through a metallic shadow mask (see Methods).

**Figure 5:**
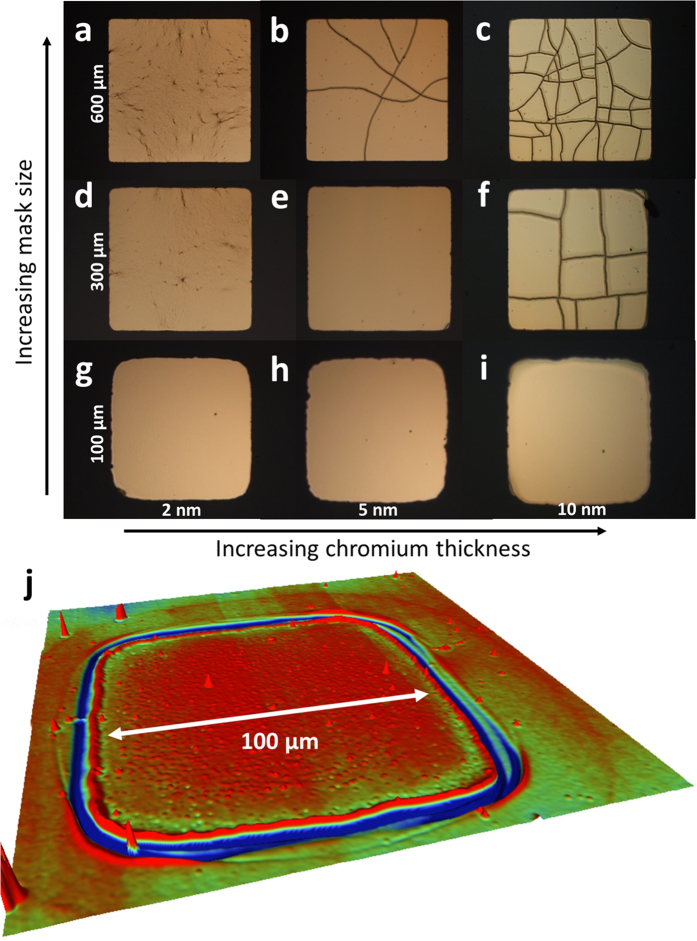
Effect of mask size and chromium thickness on the mud-crack patterning. Film thicknesses: first column (**a**,**d**,**g**) – chromium/gold = 2 nm/100 nm, second column (**b**,**e**,**h)** – chromium/gold = 5 nm/100 nm and third column **(c**,**f**,**i)** – chromium/gold = 10 nm/100 nm. Mask sizes: first row (**a**–**c**) = 600 μm × 600 μm, second row (**d**–**f)** = 300 μm × 300 μm and third row (**g**–**i)** = 100 μm × 100 μm. *D* = 1500 J. (**j**) a 3D optical profile image of the mesa feature following removal of the chromium/gold thin film.

Let us first consider square shadow masks. Figure 5 shows the influence of mask size and the chromium thickness on the metallization-induced mud-crack patterning of the PDMS surfaces. Three thicknesses of chromium/gold thin films were used: 2 nm/100 nm (Fig. 5a,d,g), 5 nm/100 nm (Fig. 5b,e,h) and 10 nm/100 nm (Fig. 5c,f,i). The size of the squares is: 600 × 600 μm^2^ (Fig. 5a–c), 300 × 300 μm^2^ (Fig. 5d–f) and 100 × 100 μm^2^ (Fig. 5g–i).

Firstly, for the 2 nm thick chromium films, cracking was not observed for any mask size – although crack-initiation sites are visible in the largest two masks (Fig. 5a,d) but not for the 100 × 100 μm^2^ masking (Fig. 5g), where a uniform film is apparent on every square. This suggests the existence of a slight influence of the pattern dimension on the internal stress state of the chromium film. Secondly, turning now to the two thicker chromium films (5 nm and 10 nm), the masking clearly has an influence over whether or not cracks are present in the metallized portion of the PDMS surface. Cracks are observed within both the 5 nm/100 nm (Fig. 5b) and the 10 nm/100 nm (Fig. 5c) 600 × 600 μm^2^ squares – even if the value of *N* is clearly lower in the 5 nm/100 nm film – in agreement with Fig. 4e.

In terms of the 300 × 300 μm^2^ squares, no cracking is apparent for the 5 nm/100 nm chromium/gold films (Fig. 5e). In contrast, for the 10 nm/100 nm chromium/gold thin films, there is always cracking in the 300 × 300 μm^2^ squares (Fig. 5f). These observations can be analysed with regard to the characteristic mud-crack pattern lengths *L*_*c*_ shown in Fig. 4b,c for the 5 and 10 nm thick chromium evaporated layers. A 10 nm chromium layer combined with a 1.5 kJ oxygen plasma dose at 40 Pa would crack for a metallization surface around 100 × 100 μm^2^ (including the standard deviation) while a 5 nm thick one would crack for a metallization surface around 230 × 230 μm^2^ (including the standard deviation). Thus, as expected, the 300 × 300 μm^2^ - 10 nm/100 nm Cr/Au – patterns are cracked but the 300 × 300 μm^2^ - 5 nm/100 nm Cr/Au – patterns remains crack-free whereas the metallized surface is slightly bigger that the expected critical one. Two alternative explanations can be suggested: (1) the degree of precision obtained on the *L*_*c*_ length estimation (for the large surface metallizations) remains too low to obtain a perfect matching between the data or (2) the characteristic mud-crack pattern length is slightly affected by the boundary condition (as for the 2 nm thick chromium films – see Fig. 5a,d,g), especially the proximity to mask boundary and a more intrinsic parameter (e.g. size and shape related) should be considered to predict cracking irrespective of mask shape. A more in-depth investigation would be necessary to clarify this question.

In terms of the 100 × 100 μm^2^ masking, no cracks are visible for 5 nm and 10 nm of chromium (Fig. 5h,i). This point seems to confirm that when the square mask dimension decreases below the characteristic mud-crack pattern lengths *L*_*c*_ – about 100 × 100 μm^2^ (including the standard deviation) for the 10 nm Cr layer – cracks are not generated anymore. Note also that Fig. 5f clearly indicates that the perpendicular nature of the cracks near to the metallization boundary is beginning to impose itself on the overall patterning of the metallized area – resulting in cracks which are perpendicular to the side of the square. In other words, the mask shape and size is leading to self-similar shapes of mesas within the metallized zone – in contrast to the polygonal shape mesas observed when large surface metallization is used – *cf*. Figs 2 and 4. Another important observation here is that we are able to form single mesa features when the metallization size is less than *L*_*c*_. Figure 5j shows a 3D optical image of such a mesa feature (100 μm by 100 μm) on the surface of the PDMS surrounded by a single crack. We demonstrate here that by progressively decreasing the mask size – from blanket metallization (see Fig. 2) to micrometre features (see Fig. 5), e.g. 100 × 100 μm squares – one moves from blanket self-organized mud-crack pattern to a single controlled crack governed by mask shape.

**Figure 6:**
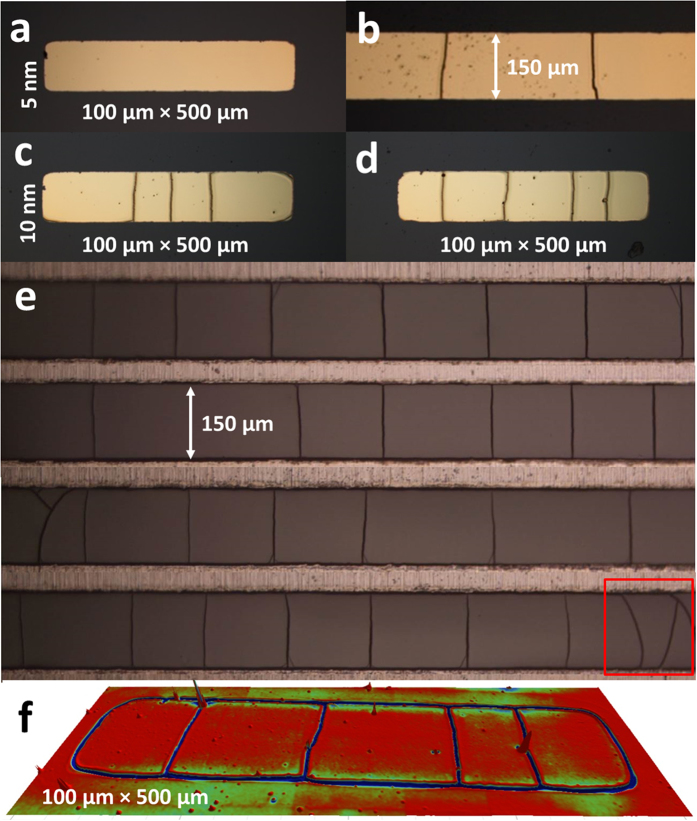
Effect of mask aspect ratio on the mud-crack patterning. (**a**) chromium/gold (5 nm/100nm) – 100 × 500 μm^2^ rectangle. (**b**) chromium/gold (5 nm/100nm) – 150 μm × 1000 μm lines. (**c**,**d**) chromium/gold (10 nm/100nm) – 100 × 500 μm^2^ rectangle. (**e**) chromium lines (150 μm × 1000 μm) following the removal of the 100 nm gold layer and (**f**) 3D image of cracking of the PDMS surface following removal of the chromium/gold (10 nm/100 nm) layer – 100 μm × 500 μm lines.

Let us now focus on metallized rectangles and lines, i.e. when the mask width is lower than *L*_*c*_ while the mask length is significantly longer. Figure 6 shows the resulting cracking of the metallized PDMS surfaces in the case of metallization of rectangles (100 × 500 μm^2^) and lines (150 μm by 1 mm). Two different thickness of chromium are used: 5 nm (Fig. 6a,b) and 10 nm (Fig. 6c–f). The gold has been removed (see Methods) in Fig. 6e to reveal the chromium and the chromium/gold has been removed (see Methods) in Fig. 6f to reveal the PDMS topography.

In the case of 5 nm of chromium, no cracks were observed in any of the smallest rectangles – Fig. 6a. However, cracks orthogonal to the metallization boundary are observed in the lines having a thickness of 150 μm – Fig. 6b – the spacing of these cracks is 296.3 ± 112.4 μm. We note that this value is, on average, larger than the value of *L*_*c*_ (217.2 ± 13 μm) obtained for the same chromium thickness and a large surface metallization. Nevertheless, the average crack-free surface obtained for the 150 × 1000 μm^2^ lines is 4.4 ± 1.7 × 10^4^ μm^2^ (i.e. 150 × 296 ± 112 μm^2^) while the value of 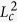 obtained for Cr/Au (5 nm/100 nm) blanket metallization is 4.7 ± 0.6 × 10^4^ μm^2^ – indicating that the crack-free surface is almost conserved irrespective of mask shape – contrary to the value of the crack spacing. Considering the 100 × 500 μm^2^ crack-free rectangles (Fig. 6a), the surface is 5 × 10^4^ μm^2^, i.e. approximately the average crack-free surface calculated above for two extreme cases (large surface and 100 × 100 μm^2^ square metallizations) – this reinforces the idea that under a characteristic surface, governed by the chromium thickness, cracks are not generated anymore in metallized features.

In the case of 10 nm of chromium, cracking is observed in all mask features (Fig. 6c–f). As with the 5 nm films, the cracks are perpendicular and straight relative to the longest mask boundary and span the smallest dimension of the mask. The cracks are relatively regularly spaced; in the case of the 100 × 500 μm^2^ rectangles – the crack spacing *L*_*c*_ = 111.2 ± 43.1 μm and the crack-free surface is 1.1 ± 0.4 × 10^4^ μm^2^ (Fig. 6d); and in the case of the 150 μm wide lines – *L*_*c*_ = 168.8 ± 33.1 μm and the crack-free surface equals 2.5 ± 0.5 × 10^4^ μm^2^ (Fig. 6e). Considering the crack-free surface measured on large surface metallization for 10 nm thick chromium films, i.e. 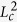 = 0.9 ± 0.1 × 10^4^ μm^2^ (see Fig. 4f), one notes that the surface is almost conserved for the 100 × 500 μm^2^ rectangles but the 150 × 1000 μm^2^ lines do not follow this rule.

In other words, the idea of an intrinsic crack-free surface domain, depending on the chromium thickness and irrespective of the pattern shape and dimension, is qualitatively consistent for the major part of results. Nevertheless, some deviations from this rule remain unexplained and would require a deeper investigation before such a method could be used in real technological applications.

By removing the chromium/gold metallization (see Methods), we observe the formation of relatively regular mesa features – as demonstrated in Fig. 6f. The figure shows a 3D image obtained using optical profiling of the cracked PDMS surface caused by the metallization shown in Fig. 6d. It can be noted that the cracking, which is parallel to the metallization boundary, is now apparent – the profile and dimensions of these cracks are comparable to the orthogonal cracks. This strengthens the argument that the silica-like PDMS cracking is caused by residual tensile stresses in the metallization as cracking is also present at the metallization boundary.

Finally, taking into account our understanding of the influence of the chromium thickness, plasma dose and mask size on the characteristic crack-free mesa length (i.e. the crack spacing), and based on the observations and qualitative analysis done on Fig. 6 – we can suggest potential routes for optimizing the cracking regularity. Firstly, by comparing lines of similar dimensions and different chromium thicknesses one observes that the uncertainty in *L*_*c*_ decreases form ~40% to ~30% as the chromium thickness increases from 5 nm to 10 nm. This implies that by optimising the residual stress (chromium thickness) in the metallization, an improved regularity of the crack spacing may be achieved. However, if the chromium thickness is increased further, then the linewidth (proximity of the boundaries) needs to be reduced to keep the cracks parallel – see red rectangle in Fig. 6e. Secondly, by comparing the standard deviations obtained for different plasma doses (see the section **Plasma-induced crack spacing**) one observes that the uncertainty in *L*_*c*_ also slightly decreases from ~7% to ~5% as the plasma dose decreases. As a consequence, by finding the appropriate combination of residual stress (metallization thickness), plasma dose and metallization shape and dimensions (proximity of boundaries) we believe that the regularity of the cracking (spacing, length and parallelism) can be optimized. However, one has to keep in mind that the stochastic nature of the cracking does not currently allow one to impose the cracking layout. Indeed, Fig 6c,d are interesting to compare as they are the same dimensions and chromium thickness but lead to two different crack patterns – 4 mesas and 5 mesas. Possible explanations for this are mask and surface defects – which could lead to notch-induced^30^,^31^ cracking. But, it is also possible that there are multiple solutions for the cracking periodicity. For example, nature provides some beautiful examples of highly periodic cracking, e.g. the Giant’s Causeway in Ireland.^4^

### Residual stress modelling and crack spacing

Attempting to physically explain the observations, i.e. the link between metal thickness, residual tensile stress and crack spacing, is not trivial. By looking at the published literature concerning experimental thin film technological cracking, it becomes clear that the ratio *λ*/*h* (with *λ the crack spacing and h the film thickness*) falls into two categories: *λ*/*h* ≫100^11^,^12^,^13^,^14^,^15^,^16^,^17^,^18^,^19^,^20^,^22^ *and λ*/*h* ≤ *100*^9^,^10^,^23^,^24^,^33^,^60^,^62^ – the observations here belong to the latter category. Crack spacing of cracked thin films has been modelled for some time now^1^,^9^,^12^,^78^,^79^,^80^,^81^,^82^,^83^,^84^,^85^. Kappert *et al*.^24^ recently reported – using examples of experimental data in the literature – that existing models are not able to predict the large *λ*/*h* ratios observed in many systems.

**Figure 7:**
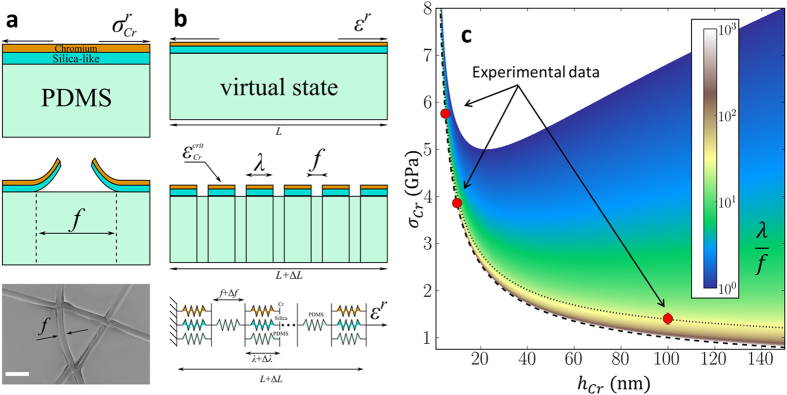
Modelling the mud-cracking of metallized, oxygen plasma-treated PDMS. (**a**) schematic diagram showing film having residual tensile stress 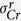 (top) and cracked film having crack width *f* (middle), and SEM image of a cracked PDMS/SiO_x_/Cr film – scale bar = 5 μm (bottom). (**b**) schematic diagram showing pre-cracked, virtual state (top) and cracked state (middle), and mechanical model of the multi-layer (bottom). (**c**) modelling of crack spacing-to-crack width ratio (*λ*/*f*) as a function of chromium thickness 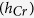 and chromium stress level 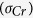. Experimental values are shown as red circles for 5 nm, 10 nm and 100 nm thick chromium films. The dashed lines are explained in the text.

In an effort to understand the cracking behaviour we develop here a simple analytical model to relate the cracking to the residual tensile stress in the layers. The details and assumptions of the model can be found in Section 6 of the Supplementary Information. We will now summarize the key elements of the model. The experimental results (see the SEM image in Fig. 7a) indicate cracking and delamination at the mesa boundaries. It is important to note that the SEM image indicates that the silica-like layer is also cracked and delaminated from the PDMS surface (not visible here) – this is in agreement with Yang *et al*.^65^ who demonstrated that the stiffness inside the cracks of plasma-oxidized PDMS is significantly less than on the non-cracked surface. This enables us to define two dimensions: *λ* (the characteristic mesa size) and *f* (the characteristic crack width) – see the schematic diagrams in Fig. 7a,b. In a first approximation the cracked layer can thus be considered to be a number of mesas composed of PDMS/SiO_x_/Cr tri-layers of size *λ* separated only by PDMS parts of size *f*.

With reference to Fig. 7b, we assume that the cracked multilayer can be represented using a one dimensional model containing a series of PDMS/SiO_x_/Cr perfectly bonded, layered mesas connected by PDMS and that a new crack occurs, within a PDMS/SiO_x_/Cr mesa, if the mesa strain reaches the critical strain of the most brittle layer (in our case, the chromium). We also assume that the residual tensile chromium stress acts as a global sample strain loading and that the multilayer transforms spontaneously from a virtual, crack free, highly deformed state (see Fig. 7b) to a state where it is composed of *N + 1* PDMS/SiO_x_/Cr mesas of size *λ* and *N* PDMS parts of size *f*. The mechanical model is shown in the bottom image of Fig. 7b. Under such assumptions one can obtain the following equation relating the crack spacing-to-crack width ratio to the stress level in the chromium film 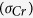 using the mechanical properties of the individual layers.


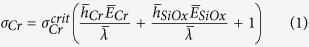


where 
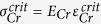
 is the ultimate tensile stress of the chromium film, 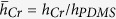 and 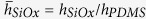 are the thickness ratios, 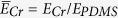 and 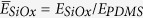 are the Young’s modulus ratios and 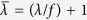 is related to the crack spacing-to-crack width ratio. There are three points to note concerning equation (1): (i) evidently the residual tensile stress must be greater than the ultimate tensile stress of the chromium to cause cracking (ii) the minimum stress level to cause cracking will be determined by the ratio of *h*_*film*_*E*_*film*_ to *h*_PDMS_*E*_PDMS_ and finally (iii) the higher the stress loading 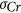 the smaller the mesa dimension *λ* and the higher the mesa density *N*. If all the individual mechanical and dimensional properties are known, equation (1) allows one to calculate the stress loading required to obtain a characteristic crack spacing *λ*. The experimental observations here allow us to have values of *λ* for different film thicknesses – the mechanical properties of the specific films (Cr, SiO_x_ and PDMS) can be found in the literature.

Let us now look at the literature to obtain experimental properties for the silica-like layer. According to the literature, there exists no general consensus concerning the thickness and the stiffness of the silica-like PDMS crust for a specific oxygen plasma dose. Indeed, the literature indicates that the stiffness, *E*_SiOx_, can range from 10 MPa to 70 GPa^19^,^56^,^62^,^64^,^65^,^86^ while the thickness, *h*_SiOx_, can range from 5 to 200 nm^10^,^55^,^86^. According to the oxygen plasma dose and pressure investigated here it is reasonable to assume that *E*_SiOx_ is of the order of 100 MPa – based on recent work by Yang *et al*.^65^ where exactly the same plasma dose has been investigated, i.e. 50 W/30 s/300 mTorr. In addition, according to recent work by Befahy *et al*.^86^ and Bayley *et al*.^55^ we can assume that the thickness of the silica-like layer is of the order of 15 nm – certainly less than 40 nm^60^. Considering the fracture of such a silica-like layer, the literature provides values of 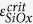 (silica and silica-like materials) ranging from 2% to 7% depending on the thickness^9^,^19^,^87^,^88^. One can assume that the critical strain, that the 15 nm thick SiO_x_ layer can sustain, is lower than 2.5% – in agreement with Coclite *et al*.^88^ concerning a 25 nm thick SiO_x_ layer. Finally, according to Huh *et al*.^28^ and Kim *et al*.^31^, and from our own observations, it seems reasonable to assume that the characteristic relaxed crack width *f* obtained by stretching silica-like PDMS or deposited SiO_x_ on PDMS ranges from 600 nm to 800 nm. The value of *E*_PDMS_ is taken to be 2 MPa^89^.

Let us now look at the literature to obtain experimental properties for the chromium. Concerning the critical failure strain (ultimate tensile strain) of very thin chromium layers (<100 nm), studies are few but Cordill *et al*.^53^ and Jin *et al*.^90^ report that 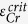 varies from ~0.2% to 2% depending on the chromium thickness (500–15 nm)^53^,^90^. Thus, the weakest layer in the SiO_x_/Cr bilayer is the chromium, and its critical strain value will be the one used as the mesa splitting criterion. In terms of the Young’s modulus of very thin chromium films (<100 nm), Petersen and Guarnieri^91^ have shown that 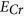 is approximatively two thirds of the bulk value for 15 nm thick chromium films, i.e. 180 GPa^91^ instead of 280 GPa^92^ for bulk chromium. Whiting and Angadi^93^ showed that the Young’s modulus increases from ~240 to ~260 GPa over a chromium thickness range of 85–300 nm. This trend follows the model given by Sun and Zhang^94^ which predicts a significant fall in the value of Young’s modulus under a critical thickness value. These experimental values of 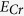 and 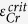 are plotted as a function of chromium thickness in Supplementary Fig. 7 of the Supplementary Information. Firstly, we observe that in the case of thin chromium the critical thickness below which the stiffness and critical failure strain change rapidly is of the order of 100 nm. Secondly, the experimental data can be fitted by analytical expressions – 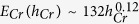 and 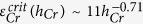 where 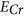 is in GPa, 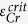 is in % and 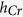 is in nm. These analytical fits can be used with equation (1) above to calculate the level of residual stress in the chromium film which leads to experimental film cracking.

Let us now apply these published material properties to equation (1). An initial qualitative analysis, using the assumed values available from the literature, indicates that first term in brackets in equation (1) is of the order of 10^−2^ and the second term in brackets is of the order of 10^−6^. Thus, it is clear that for the PDMS/SiO_x_/Cr system used here the influence of the silica-like layer is several orders of magnitude smaller than the influence of the chromium – this is in good agreement with the results presented in Fig. 4e–g.

Based on equation (1) above, the experimental values chosen from the literature and the behaviour of thin chromium films (see Supplementary Fig. 7 in the Supplementary Information), Fig. 7c shows a plot of *λ*/*f* obtained by varying 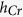 and a loading stress 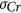. Also plotted on Fig. 7c are the experimental values (red circles) of *λ*/*f* (for *f* = 700 nm) for 5 nm, 10 nm and 100 nm chromium film thickness at an oxygen plasma dose of 1.5 kJ (see Fig. 4).

Let us now discuss Fig. 7c. Two limit cases are apparent from the figure: (i) a non-cracked to cracked boundary (lower envelope) indicated by a long dashed line – this corresponds to the case where the crack spacing-to-crack width 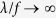 and 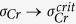, and (ii) a maximum cracked state (upper envelope) where 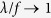 . Focusing first on the lower envelope, we observe that the thinner the chromium film, the higher the required stress value to crack the film. This is due to the mechanical softening of thin (<100 nm) chromium films (see Supplementary Fig. 7 in the Supplementary Information) observed in the literature. One observes, for example, that a ~1 GPa stress loading is necessary to fracture a 100 nm thick chromium film while ~10 GPa would be necessary to fracture a 2 nm thick chromium film. Next, one observes that the thicker the chromium film, the higher the stress level that the chromium film can sustain before reaching the upper (envelope) limit case, i.e. its maximum cracked state. Indeed, a 100 nm thick chromium film cracks at low stress but would require ~6 GPa of *additional* stress to reach the upper limit. In contrast, a 2 nm thick chromium film would crack at a higher stress but would reach the upper limit case with very little additional stress. In practice, no cracks are observed using a 2 nm thick chromium film (see Figs 4 and 5) suggesting that the value of the residual tensile stress is less than 10 GPa. In addition, irrespective of the chromium thickness, the maximum cracked state 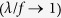 is never observed experimentally. This implies that the residual tensile stresses in the evaporated 5 nm, 10 nm and 100 nm films are in principle less than 6.5 GPa, 5.5 GPa and 6.8 GPa respectively.

By considering a fixed chromium thickness, we can look at crack generation between the two limit cases. The higher the stress loading (with 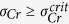 ), the smaller the value of *λ*/*f*, i.e. the crack spacing reduces for a given crack width *f*. In addition, close to the chromium critical tensile stress, a small increase in the loading stress leads to a large decrease in the crack spacing whilst far from the critical tensile stress, a large increase in the loading stress is required to cause new cracks. In the context of the model, this phenomenon is due to the fact that every new crack creates an additional soft (highly stretchable) zone that leads to stress relaxation within every individual metallized mesa.

Let us now discuss the experimental data points (red circles in Fig. 7c) in the context of the model. One observes that the experimental points are close to the lower envelope 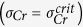 but slightly deviate from it as the chromium thickness increases. The residual tensile stress (short dotted line data fit) reduces as the chromium thickness increases – 5.8 GPa (at 5 nm), 3.9 GPa (at 10 nm) and to 1.5 GPa (at 100 nm). These levels of residual tensile stresses in the chromium films are significant and imply that the multi-layer is subjected to strains of 3.6% (at 5 nm), 2.2% (at 10 nm) and 0.6% (at 100 nm) during the metallization – very close to the critical failure strains of each film (see Supplementary Fig. 7 in the Supplementary Information). The estimated values of residual tensile stress and the trend are in very good agreement with the litterature^51^,^52^,^53^ which shows that the residual stress in chromium films decreases exponentially from >3 GPa to 800 MPa over the thickness range 15–500 nm. Nevertheless, we note that Berger and Pulker^51^ and Janssen and Kamminga^52^ both observed a maximum in the residual stress in the chromium film at a thickness of 15 nm and that the residual stress decreases markedly below this value reaching 1.2 ± 0.5 GPa^51^,^52^. This observation is not taken into account in our model which is based on fitted and extrapolated Young’s moduli and critical strain values for data in chromium film thicknesses greater than 15 nm. Thus, it is probable for the 5 nm thick film that the model overestimates the value of the stress which causes cracking. A better understanding of the mechanical properties of thin chromium films, not currently available in the literature, would clarify this point. To conclude this section, the model fits the experimental observations for chromium thicknesses greater than 10 nm and could be useful in predicting crack generation and spacing (in the case of 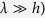 or loading stresses (residual film stresses or external loading stresses) which cause cracking in multi-layer films on flexible substrates.

### Conclusion

We demonstrate a new method to generate and control crack-based patterns achieved only using residual tensile stresses (<4 GPa) in evaporated thin chromium films (5–100 nm) deposited onto PDMS which has been exposed to oxygen plasma. Such metallization-induced cracking strategy is in contrast to methods using external loading^63^,^76^ – which requires a cumbersome setup – or the use of top-down, pre-defined notches^30^,^31^ or wet processes^95^. The density of the mud-crack patterning is controlled by tuning processing parameters: the density increases significantly with chromium film thickness and decreases slightly with oxygen plasma dose. The self-organization of the mud-crack patterning observed on large metallized surfaces can be controlled by masking, i.e. imposing specific metallization boundaries: (1) close to the characteristic mud-crack length (chromium thickness dependent), parallel and quasi-periodic cracks are spontaneously created during film evaporation, (2) below such a characteristic mud-crack length, only the masking feature boundary is cracked this allows the transfer of masking features into the PDMS. Finally, the study suggests that it will be interesting to see the limits of such organized cracking approaches by reducing the feature sizes smaller than those possible using mechanical shadow masking, e.g. by using photolithography or electron beam lithography combined with metallization.

## Methods

### PDMS processing

All chemicals used in this work were used unmodified and off-the-shelf. All processing was performed in a class ISO 5/7 cleanroom. The PDMS is a two liquid component kit – Sylgard 184 Elastomer (Dow Corning, USA) – containing the vinyl-terminated base and the curing agent (methyl hydrogen siloxane). A PDMS mixture was prepared by mixing the base and the curing agent to a mixing ratio – by weight – of 10:1. As recipients to mould PDMS sheets, commercial Teflon® coated stainless steel oven dishes (Kitchen Craft, UK) having dimension 16.5 × 10 × 1 cm were cleaned using VLSI quality acetone, IPA and deionized water followed by a dehydration bake at 165 °C. The PDMS mixtures were then poured into the dishes using a specific volume of the mixture in order to form 1 mm thick uniform films. Mixing and pouring invariably incurs the formation of trapped air bubbles in the mixture, which are removed using five successive pumping cycles to 1 mbar. The PDMS mixtures were then placed onto a level hotplate for 2 hours at 100 °C. The PDMS sheets are then carefully removed from the Teflon coated recipients and diced into samples having a surface of 1 cm^2^.

### Oxygen plasma treatment

The PDMS samples (1 cm^2^ by 1 mm) were exposed to low frequency oxygen plasma using a ‘Pico’ (Diener Electronic, Germany) barrel type, 0–200 W capacitive-coupled radio frequency discharge at 40 kHz. The plasma chamber has a volume of 4 × 10^−3^ m^3^. The PDMS samples were exposed to the oxygen plasma without a prior solvent clean. In all cases, a single PDMS sample was loaded into the chamber, the chamber was initially pumped to a pressure of <0.2 mbar and then pure oxygen (99.99%) was let into the chamber to achieve a pressure of 0.4 mbar. The plasma power (W) and exposure time (sec) was modified for different PDMS samples to enable the sample to be exposed to a certain plasma dose *D* – plasma power (W) × plasma time (s) – between 360 J and 180 kJ. The sample temperature rise during the plasma treatment was measured – see Section 2 of the Supplementary Information.

### Metallization and further processing

Following the oxygen plasma treatment, the PDMS samples were evaporated in a MEB 550S electron beam evaporation system (Plassys, France) at <10^−7^ mbar with thin films of metal chromium and gold – the chromium thickness was varied from 2 nm to 100 nm and the gold thickness was 100 nm. During evaporation, the PDMS sample is maintained at room temperature using a cooling system. The deposition rates were 0.2 nm s^−1^ and 0.5 nm s^−1^ for the chromium and the gold respectively. Great care was taking during sample handling to avoid accidental deformation-induced cracking. Our observations indicated that we could not induce cracking of the silica-like layer on pre-metallized PDMS – exposed to 1.5 kJ dose (0.4 mbar) oxygen plasma – either by mechanically deforming up to 50% strain or by heating to 250 °C; this is not the case at higher doses^64^. However, once metallized careless handling of the samples could result in extra cracks in the metallization. Firstly, due to the roughness of the metallic support the PDMS samples did not stick to them during the high vacuum of the evaporation. Secondly, the shadow mask was carefully removed in a liquid environment (isopropyl alcohol). Finally, the thickness of the PDMS samples (1 mm) limited sample bending due to gravity. The thin films of metal were removed using a potassium iodide (KI) based wet etch (AU-5) for the evaporated gold thin films and a ceric ammonium nitrate (NH_4_)_2_Ce(NO_3_)_6_ and concentrated perchloric acid (HClO_4_) based wet etch (CR-7) for the evaporated chromium thin films^64^. For a specific chromium thickness and plasma dose, the statistical data concerning the mean values of *N* (crack density per square metre) was obtained from two samples: large surface (blanket) metallization and the metallization using the shadow mask. The mechanical shadow mask is 50 μm thick and contains 370 features of different sizes: 2 × 2 mm (10), 1 × 1 mm (25), 600 × 600 μm (35), 300 × 300 μm (55), 100 × 100 μm (115) squares, 100 × 500 μm lines (40) and 150 μm wide lines (90).

## Additional Information

**How to cite this article**: Seghir, R. and Arscott, S. Controlled mud-crack patterning and self-organized cracking of polydimethylsiloxane elastomer surfaces. *Sci. Rep*. **5**, 14787; doi: 10.1038/srep14787 (2015).

## Supplementary Material

Supplementary Information
