# Supplementary material for: Controlled mud-crack patterning and self-organized cracking of polydimethylsiloxane elastomer surfaces
Source: Sci Rep. 2015 Oct 6;5:14787. doi: 10.1038/srep14787 (PMC4594096; doi:10.1038/srep14787)
Supplement: Supplementary Information [file srep14787-s1.pdf]

Supplementary Information for 'Controlled mud-crack patterning and self-organized cracking of polydimethylsiloxane elastomer surfaces'

Rian Seghir and Steve Arscott

Institut d'Electronique, de Microélectronique et de Nanotechnologie (IEMN), CNRS UMR8520, The University of Lille, Cité Scientifique, Avenue Poincaré, 59652 Villeneuve d'Ascq, France

Correspondence and requests for materials should be addressed to R.S and S.A. (rian.seghir@iemn-univ-lille1.fr and steve.arscott@iemn.univ-lille1.fr)

1. Oxygen plasma treatment of the PDMS

Supplementary Figure 1 illustrates the oxygen plasma treatment steps and their subsequent effects on the PDMS samples. First, the PDMS sample is loaded into the plasma chamber and the chamber is pumped down to a pressure of 0.2 mbar (20 Pa) – see Supplementary Fig. 1a. Oxygen gas is then let into the chamber at a fixed flow rate in order to achieve a chamber pressure of 0.4 mbar. The temperature of the PDMS sample is T K. The oxygen gas is then ionized by applying the RF signal; during this step presence of the plasma will cause the PDMS to heat up (T+ΔT K) and to expand according to its thermal expansion coefficient α ~3×10^{-4} K^{-1} from -55 to 150°C[1] – see Supplementary Fig. 1b. The chemical effect of the plasma is to form a silica-like layer[2,3] on the exposed surfaces of the PDMS, see Fig. Supplementary 1c at the elevated temperature; the thickness of this layer is known to depend on the plasma dose.[3,4] A silica-like crust – having residual tensile stress σ_{crust}^{vol}– is created on a pre-strained σ_{crust}^{th}piece of PDMS. Depending on the plasma dose and oxygen pressure, one of two outcomes are observed in the current work: (i) the silica-crust spontaneously cracks, relaxing the stress - see Supplementary Fig. 1d or (ii) the silica-crust does not crack but remains in residual tensile stress,[3] see Supplementary Fig. 1e. When the plasma is turned off, the temperature gradually falls to T K – with the aid of the presence of the venting gas (N_2) – and the PDMS contracts, reducing the residual tensile stress in the silica-like crust (cracked or non-

cracked) - see Supplementary Fig. 1f and Supplementary Fig. 1g. The amount of residual tensile stress present in the silica-like crust – at the end of the process - will depend on the parameters of the oxygen plasma,[3] i.e. dose and pressure; and these - as we shall see - will determine the nature of the cracking and the spontaneous cracking/non-cracking boundary.

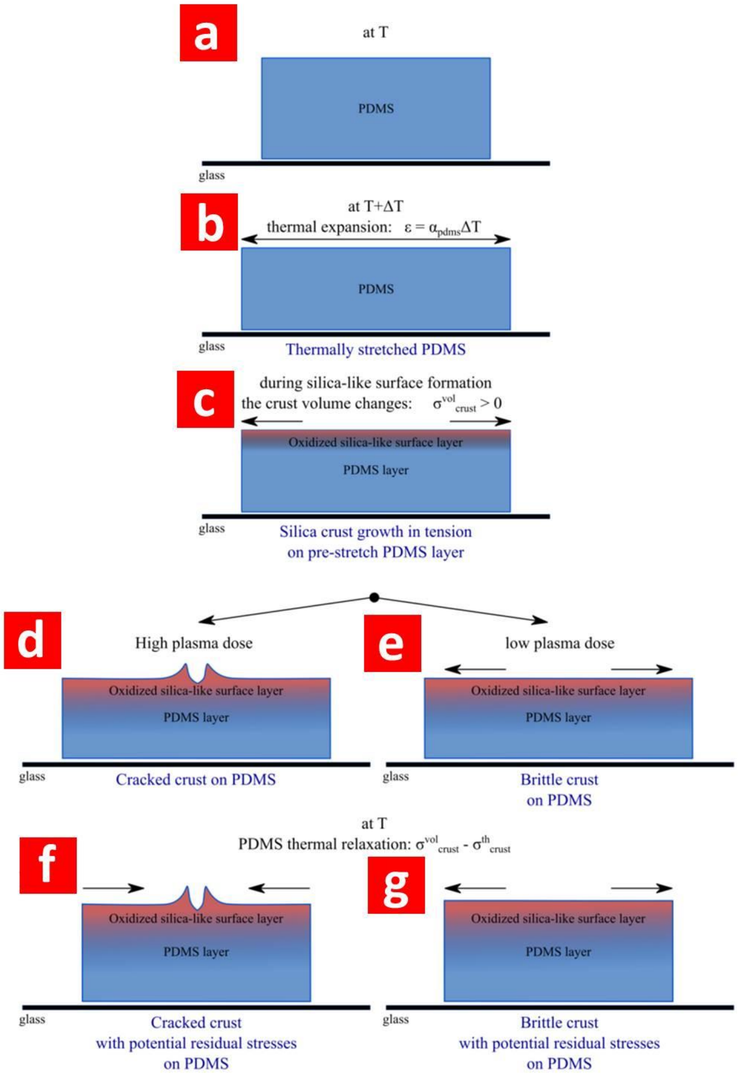

Supplementary Figure 1 Oxygen plasma treatment of the PDMS samples. a, Initial PDMS size at *T* K. **b**, expanded PDMS at *T*+Δ*T* K. **c**, creation of silica-like crust on exposed PDMS at *T*+Δ*T* K. **d**, spontaneous cracking at high dose oxygen plasma. **e**, non-cracked silica-like crust at moderate dose oxygen plasma and **f**, and **g**, contraction of PDMS and silica-like crust layer as sample returns to *T* K.

2. Thermal expansion of the PDMS during the plasma treatment

In order to evaluate the role of thermal expansion of the PDMS in the process, the temperature rise of the PDMS - during oxygen plasma - exposure was evaluated. This was achieved *ex situ* for every plasma dose (1.8 kJ – 180 kJ) after 35 seconds venting with N_2 using a piece of PDMS resting on a glass slide exposed to the plasma and using a FI622TI laser thermometer (Française d'Instrumentation, France). Supplementary Fig. 2 shows the temperature the PDMS reaches during the plasma dose. To extrapolate the actual maximum temperature *during* the plasma, we measured the heat loss of a PDMS sample as a function of temperature – see inset to Supplementary Fig. 2. For example, the PDMS sample which receives the highest dose (180 kJ at 40 Pa) reaches a temperature of 57°C during plasma treatment corresponding to a ΔT of 37°C - i.e., a strain of 1.2%. At a plasma dose of 1.5 kJ (50W/30s) the sample reaches a maximum temperature of 22.5°C corresponding to a strain of <0.1%.

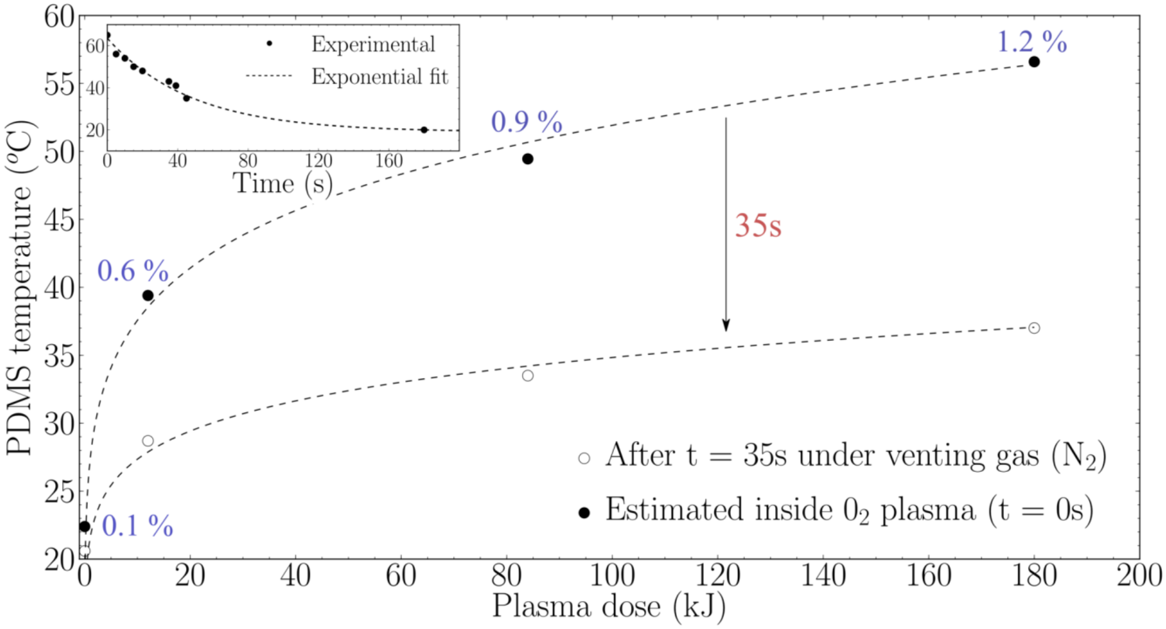

Supplementary Figure 2 PDMS sample temperature as a function of oxygen plasma dose. Inset shows cooling curve - temperature (°C) versus time (sec).

3. Spontaneous cracking of PDMS elastomer exposed to high dose, high pressure oxygen plasma

Supplementary Figure 3 shows optical microscope images of the surfaces of PDMS samples which were exposed to high pressure[5] (0.4 mbar - 40 Pa) oxygen plasma as doses in the range from 1.8 kJ to 180 kJ. In all cases, spontaneous cracking of the PDMS surfaces was observed. Over the range of doses tested, the global cracking patterning evolves from being parallel line-type cracking at lower doses (1.8 kJ – 12 kJ) – Supplementary Fig. 3a - to polygonal patterns at intermediate doses (12 kJ – 24 kJ) – Supplementary Fig. 3b-c - to polygonal patterns having curved cracks at higher doses (24 kJ – 180 kJ) – Supplementary Fig. 3d. The parallel line-type cracking resembles that observed by Tooley *et al.*[6] using thermal cycling of PDMS exposed to oxygen plasma. The cracking threshold plasma dose observed here corresponds well to that reported by Gorrn and Wagner[5] and Befahy *et al*.[7] For the lower doses, i.e. < 5 kJ, the cracking is more apparent at the edges of the samples. For higher doses, the cracking is uniform all over the 1 cm^2 PDMS surface. Interestingly, as the plasma dose is increased from 12 kJ to 180 kJ the cracking patterning is observed to become less dense, i.e. the number of polygonal mesa structures per surface N decreases. The number of mesa features per surface (i.e. the crack density) is estimated to be $9.1\pm5\times10^{9}$ m^{-2}, $2.1\pm1.2\times10^{9}$ m^{-2} and $1.6\pm1.7\times10^{9}$ m^{-2} for plasma doses of 12 kJ, 24 kJ and 180 kJ respectively. These values correspond to an average crack spacing of 10.5±2.1 µm, 22.1±4.7 µm and 25±4.5 µm.

Let us now look at the topography of the individual cracks themselves as a function of plasma dose and time. The insets to Supplementary Fig. 3a-d show the topography of the individual cracks and were obtained using a Contour GT-X 3D optical profiler (Bruker Corp., USA). It can be seen that the profile of the cracks evolve from apparent ridge-like defects (1.8 kJ – 12 kJ) to cracks having an inverted 'Mexican hat'-like profile (>12 kJ). Supplementary Figure 4 shows topographical data concerning the cracks obtained using optical profiling. Supplementary Fig. 4a shows the depth d (blue circles) and height h (red squares) of the cracks in the dose range (12 kJ – 180 kJ) and the height h of the ridges in the dose range (1.8 kJ – 12 kJ). The measurements were made 1 day after exposure to oxygen plasma. It can be seen that the vertical dimensions are a function of plasma dose – the total depth of the cracks changes from

50 nm to 300 nm over the plasma dose range 12 kJ to 180 kJ. The crack depth is a not linear function of plasma dose – a saturation depth of around 300 nm being observed at very high plasma dose. These crack dimensions are in good agreement with the thickness of the silica-like crust layer[4] and indicate that it is this layer which is cracking.

Supplementary Fig. 4b shows the evolution of the spontaneous cracking of the thin silica-like crust at PDMS surface as a function of time directly after plasma exposure – in this case the oxygen plasma dose 14.4 kJ. Supplementary Fig. 4b indicates that the cracking, i.e. the relaxation of the stress, varies exponentially with time over the first 3 hours following exposure to the oxygen plasma – cracking over this time scale was observed for all plasma doses tested. It is observed that the depth of the cracks and the width closely follow exponential curves within the data error bars - for example, for 14 kJ we have: $h = -46.5e^{-0.02t} + 57$, $d = 58.5e^{-0.02t} - 66$ and $w = 0.68e^{-0.03t} + 1.59$.

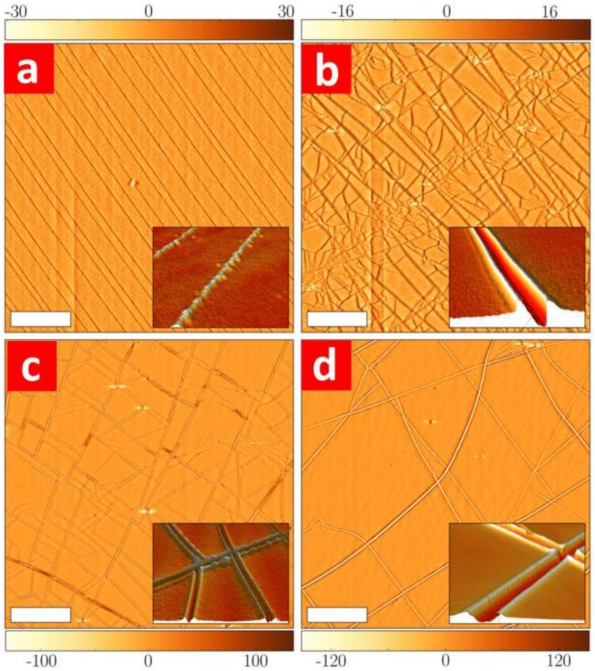

Supplementary Figure 3 Spontaneous cracking of the PDMS surface using oxygen plasma. O_2 pressure = 0.4 mbar, plasma frequency = 40 kHz. **a**, Dose *D* = 3.4 kJ, **b**, *D* = 12 kJ, **c**, *D* = 24 kJ **d**, *D* = 180 kJ (Scale bar = 50 μm and colour bar units are in nanometres), the insets show 3D profiles of the cracks (inset length = 10 μm).

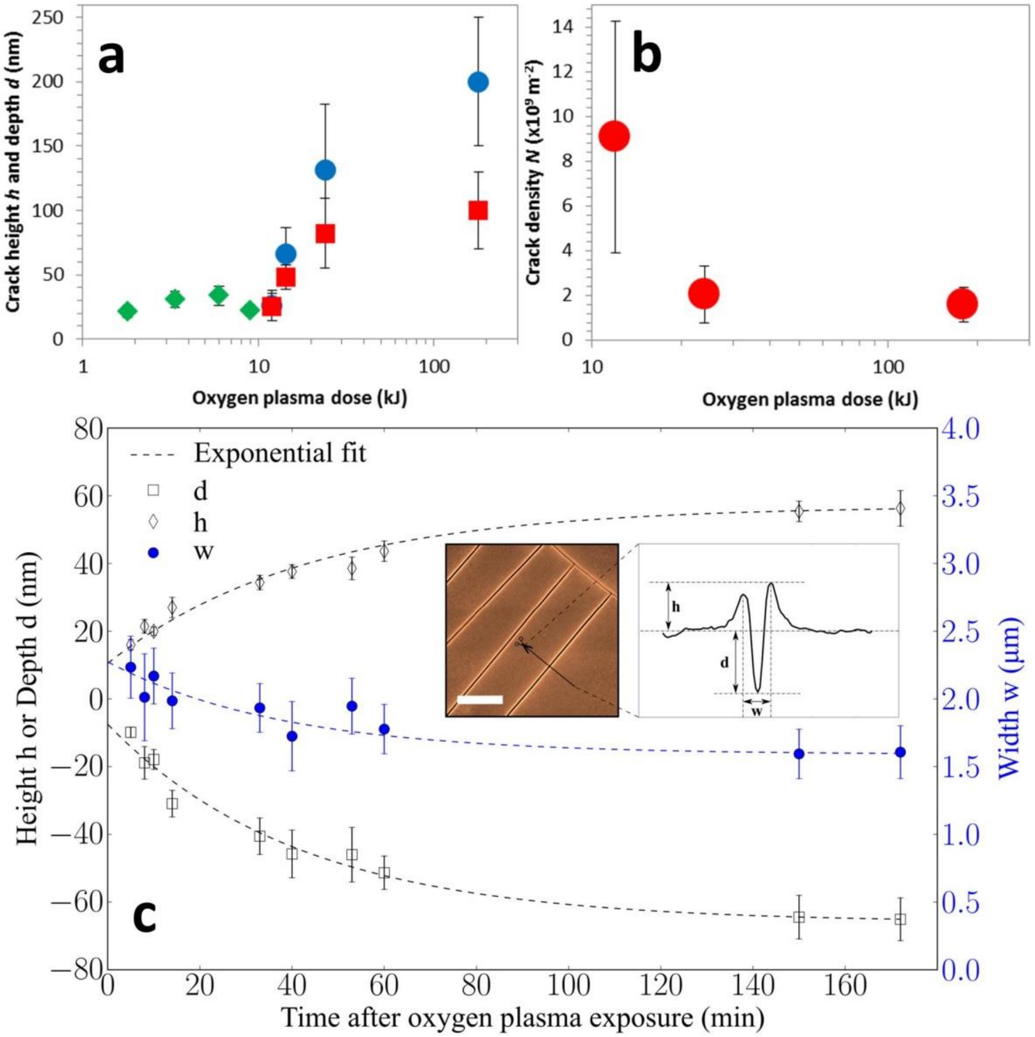

Supplementary Figure 4 Topography of the cracking of the PDMS surface induced by oxygen plasma. **a**, the height *h* (green diamonds and red squares) and depth *d* (blue circles) of the cracks as a function of plasma dose - the data was recorded 1 day after exposure to the plasma. **b**, the crack density *N* as a function of plasma dose and **c**, the height *h*, depth *d* and width *w* of a crack as a function of time for a single dose 14.4 kJ, O_2 pressure = 0.4 mbar, Plasma frequency = 40 kHz. The inset (to c) shows the dimensions of the crack profile. The height *h* of the crack (open diamonds), the depth *d* (open squares) - relative to the PDMS surface far from the crack – and the width *w* (closed circles) of the crack. The dashed lines correspond to exponential fitting of the data. The error bars are the standard deviations obtained using several profiles.

4. Wetting contact angle on PDMS as a function of plasma dose: the influence of cracking

The impact of the plasma dose on the surface energy of the PDMS was assessed using contact angle measurements. Supplementary Fig. 5 shows the wetting CA of DI water on the PDMS surfaces as a function of plasma dose. The CA were measured - Digidrop CA Meter (GBX Instruments, France) - immediately after plasma exposure (open squares) and 3 weeks later (open diamonds) - in order to study the effect of plasma dose and ageing of the surfaces. Let us first consider the immediate CA measurements. The CA of DI water on untreated PDMS is 107.3±0.8° - this value falls rapidly for low dose oxygen plasma (1.8 kJ) to 16.2±1.8°. The CA remains low (hydrophilic surface) until a dose of 9 kJ - CA = 12.4±3.2°. Interestingly, for $D > 9$ kJ, the CA increases to a saturation value of 54.8±7° at 24 kJ.

When the CA is measured 3 weeks later, the measured values are much nearer to the value of untreated PDMS. However, the data indicates that there is a minimum for the PDMS exposed to a plasma dose of 1.8 kJ. Supplementary Fig. 5 shows photographs of the cracking caused by the plasma treatment - taken using a DM4000M optical microscope (Leica AG, Germany). As the plasma dose is increased, the transition from no cracking <1.8 kJ (points 1-3) to some edge cracking around 1.8 kJ (point 4) to straight cracks between 1.8 kJ and 9 kJ (points 5-7) to polygonal patterns >10 kJ (points 8-9) is apparent. For the CAs measured straight away, higher CAs are associated with low plasma doses (no cracking) and high plasma dose (high density polygonal cracking). An oxygen plasma dose of 1.8 kJ (0.4 mbar, 40 kHz) apparently gives the most durable hydrophilic surface - CA equal to 69.7±2.5° after 3 weeks storage in air in the cleanroom. Smooth surface features are known the modify wetting and contact angle hysteresis[8,9] - this could explain here the elevated contact angle for high dose plasma. It is important to note that although the CAs vary with time, the crack features on the surfaces of the PDMS samples are still apparent after 3 weeks – this indicates that the CA changes are due to a modification of the surfaces not the topography of the cracks.

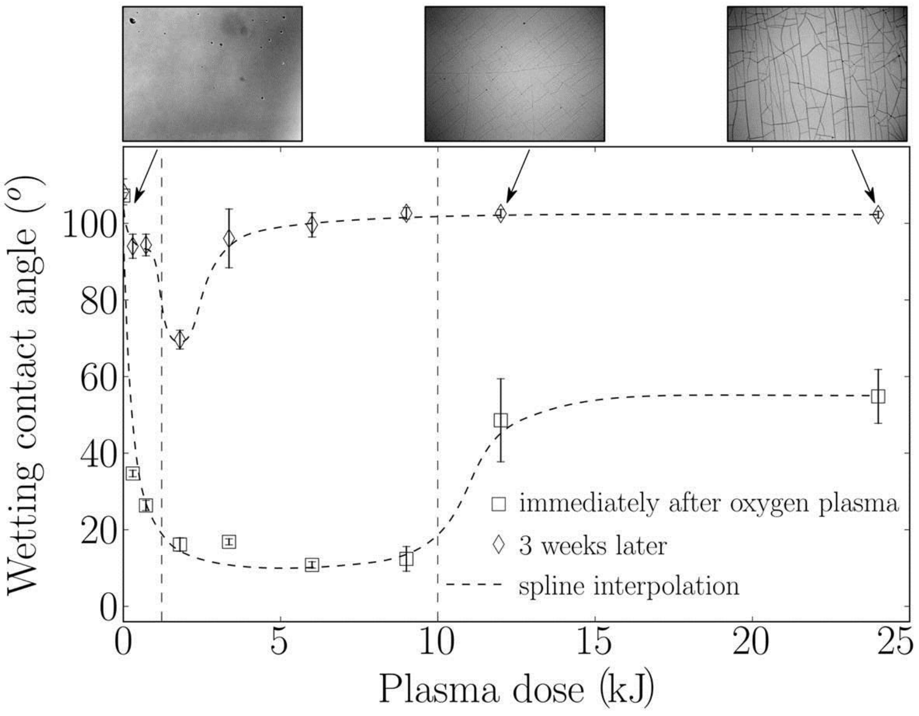

Supplementary Figure 5 Wetting contact angle of deionized wafer on the PDMS surface as a function of oxygen plasma dose. Inset shows photographs of the PDMS surface immediately after oxygen plasma.

5. Atomic force microscopy (AFM) measurements of the PDMS surfaces

Tapping mode atomic force microscopy (AFM) measurements were conducted on the PDMS surfaces using a Dimension D3100 (Bruker-Veeco USA). Over the plasma dose range and oxygen pressure studied here, the AFM did not reveal the presence of organized winkles[1,10] known to form on PDMS surfaces following exposure to low dose O_2 plasmas. No spontaneous cracking of the PDMS surfaces is observed following exposure to oxygen plasma doses less than 1.8 kJ. Indeed, the PDMS surfaces exposed to a 40 Pa pressure oxygen plasma over the dose range 360 J to 1.5 kJ are flat – as observed using AFM. Our experiments indicate that PDMS surfaces exposed to oxygen plasma in the dose range < 1.8 kJ are stable indefinitely and do not crack – samples kept for weeks do not demonstrate spontaneous cracking and their surfaces remain smooth. The PDMS surfaces between spontaneous cracks (D >1.8 kJ) are flat – see

Supplementary Figure 6a. AFM measurements on the cracks are consistent with the finding of the 3D optical profiling – see Supplementary Figure 6b.

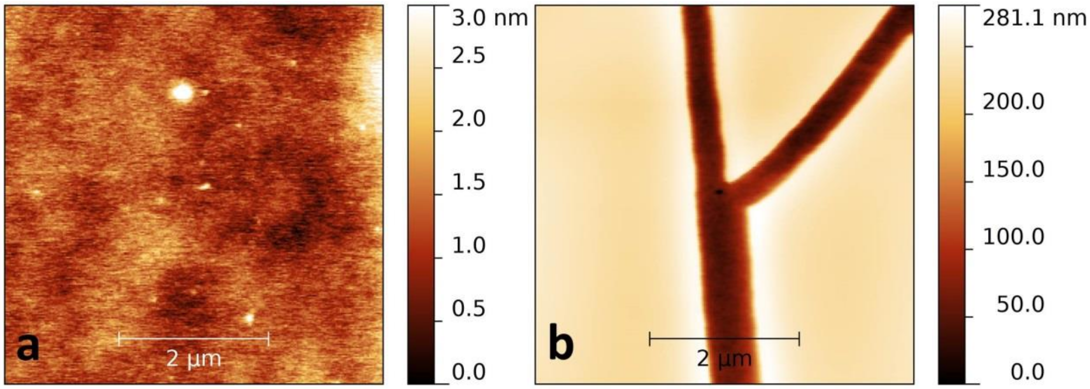

Supplementary Figure 6 Atomic force microscopy measurements. **a**, PDMS surface between cracks. **b**, a crack generated using an oxygen plasma dose of 24 kJ.

6. Modelling of the residual stress in the chromium film

This section describes the analytical model used here to estimate the level of residual stress in an evaporated chromium layer of thickness h deposited on an oxygen plasma exposed PDMS sample.

As detailed in the text, the evaporated gold layer does not affect the mesa radius λ but only contributes to increasing the crack width by providing additional stresses on pre-cracked mesas. Evaporated gold films are known to have residual tensile stresses much less than that of evaporated chromium films on polymeric substrates[11] - thus, the gold layer is not considered in model. Consequently the modelled multilayer is composed of a thick and flexible PDMS substrate onto which two highly brittle thin layers are well adhered: a thin layer of silica-like PDMS and a thin layer of chromium. These thin films are modelled by their elastic moduli E_{SiOx} and E_{Cr}, their thicknesses h_{SiOx} and h_{Cr} and their critical strains before brittle failure $\varepsilon_{SiOx}^{crit}$ and ε_{Cr}^{crit}. According to SEM image in Fig. 7a of the manuscript, experimental cracks reveal a partial

delamination of the chromium/silica-like PDMS over a limited domain noted *f*. We assume consequently that the cracked multilayer can be modelled using a 1D model composed of a series of PDMS/SiO_x/Cr perfectly bonded layers of size λ, connected with PDMS of size *f* and that a new crack occurs, within a PDMS/SiO_x/Cr mesa, if the mesa strain reaches the critical strain of the weakest mesa layer. Finally, we assume that residual tensile chromium stresses act as a global sample strain loading and that the multilayer changes spontaneously from a virtual, crack free, highly deformed state to a state where is it composed of *N+1* PDMS/SiO_x/Cr mesas of size λ and *N* PDMS mesas of size *f*. The meaning of the symbols in the below equations are given in Fig. 7a and Fig. 7b of the manuscript. The sum of elongation along the loading direction gives:

$$\Delta L = (N+1)\Delta\lambda + N\Delta f \tag{1}$$

$$(N+1)\lambda + Ndf = L \tag{2}$$

$$\Delta L = L\varepsilon_{total} \tag{3}$$

$$\Delta\lambda = \lambda\varepsilon_{mesa} \tag{4}$$

$$\Delta f = f\varepsilon_{crack} \tag{5}$$

For layers (l) in series, the force is the same in individual parts; for layers in parallel, the strain is the same in the individual parts. Thus, it follows that:

$$\varepsilon_{crack} = \varepsilon_{mesa}\frac{\sum_{l=0}^{n} h_l\, E_l}{h_{crack}E_{crack}} = \varepsilon_{mesa}\frac{h_{pdms}E_{pdms} + h_{SiOx}E_{SiOx} + h_{Cr}E_{Cr}}{h_{crack}E_{crack}} \tag{6}$$

We can denote $M = \frac{\varepsilon_{mesa}^{crit}}{\varepsilon_{total}}$ to be the ratio between the critical mesa strain and the global strain and $S = \frac{\sum_{l=0}^{n} h_l E_l}{h_{crack}E_{crack}}$ to be the ratio between the equivalent mesa stiffness and the crack region stiffness. One obtains:

$$\lambda = \frac{MS-1}{L^{-1}(MS-1) + f^{-1}(1-M)} \tag{7}$$

In the following expression, we see that the characteristic length λ is function of three physical

quantities: (1) the ratio between the strain (or stress) amplitude and the critical mesa strain (or stress), (2) the ratio between the equivalent stiffness of the metallized mesas and of the crack domains, (3) the metallization and crack dimensions. Let us consider the case, where L tends toward infinity, i.e. large metallization or infinite metallized lines. In this case equation 1 becomes:

$$\frac{\lambda}{f} \approx \frac{MS - 1}{1 - M} \tag{8}$$

where $\frac{\lambda}{f}$ is a geometrical parameter, i.e. the crack spacing-to-crack width ratio.

If we now focus on the specific practical case presented in the paper, i.e. the PDMS/SiO_x/Cr system, this general equation can be rewritten to explicitly relate the chromium stress loading, the crack spacing and the mechanical properties of the materials to give:

$$\sigma_{Cr} = \sigma_{Cr}^{crit} \left(\frac{\bar{h}_{Cr}\bar{E}_{Cr}}{\bar{\lambda}} + \frac{\bar{h}_{SiOx}\bar{E}_{SiOx}}{\bar{\lambda}} + 1 \right) \tag{9}$$

where $\sigma_{Cr}^{crit} = E_{Cr}\varepsilon_{Cr}^{crit}$ is the ultimate tensile stress of the chromium film, $\bar{h}_{Cr} = h_{Cr}/h_{PDMS}$ and $\bar{h}_{SiOx} = h_{SiOx}/h_{PDMS}$ are the thickness ratios, $\bar{E}_{Cr} = E_{Cr}/E_{PDMS}$ and $\bar{E}_{SiOx} = E_{SiOx}/E_{PDMS}$ are the Young's modulus ratios and $\bar{\lambda} = (\lambda/f) + 1$ is related to the crack spacing-to-crack width ratio. This is the equation presented in the manuscript. Nevertheless, as explained in the manuscript, according to values in the literature, the second term in brackets in the above equation (i.e. the contribution of the SiO_x) can be neglected for low plasma doses to give the following simplified expression for the level of the residual tensile stress in the evaporated chromium film:

$$\sigma_{Cr}^{r} = \sigma_{Cr}^{crit} \frac{\bar{h}_{Cr}\bar{E}_{Cr}}{\bar{\lambda}} + \sigma_{Cr}^{crit} \tag{10}$$

7. Young's modulus and critical failure strain of thin chromium films

Supplementary Figure 7 below shows a plot of the experimental values available in the literature of the Young's modulus[12–14] and the critical failure strain[15–17] of thin chromium films in the range 15-500 nm.

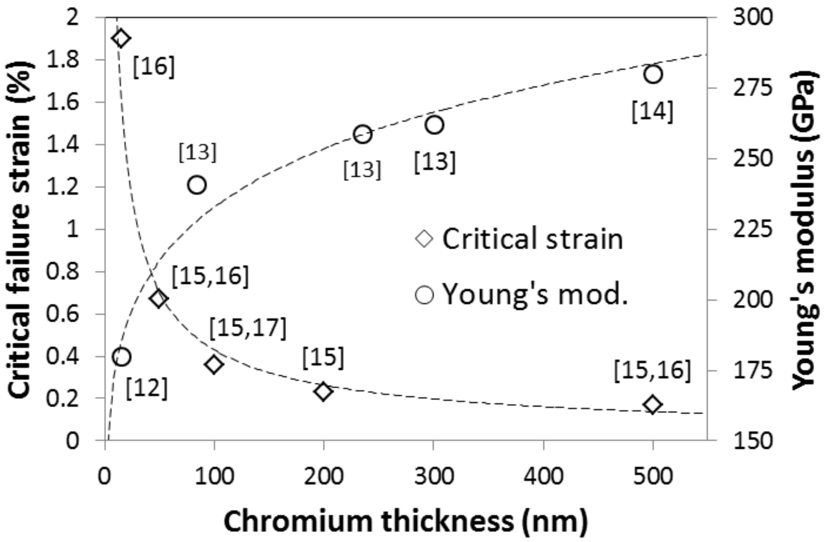

Supplementary Figure 7 Plot of the experimental values available in the literature of the Young's modulus and the critical failure strain of thin chromium films. Open circles = Young's modulus, open diamonds = critical failure strain. The data labels correspond to references (see the reference list below). The dashed lines correspond to power law fits given and used in the manuscript.

One should note that the mechanical properties of thin films can be affected by their uniformity. It is well known that metal-insulator percolation transition for very thin evaporated films is about 2 nm for chromium[18] and between 6 nm and 20 nm for gold[19] depending on processing conditions, e.g. vacuum pressure, deposition rate and substrate temperature.[20] This is the reason why we chose the chromium thickness to be in the range 2-100 nm and the gold thickness to be fixed at 100 nm.

Acknowledgements (Supplementary Information)

The work was performed within the *Laser Processing Platform for Multifunctional Electronics on Flex* (LEAF - ANR-11-EQPX-0025) EQUIPEX project – funded by the French Government. The authors thank Dr Emmanuel Dubois (Head of the LEAF project) for the use of equipment purchased within the project. The authors also thank Dominique Deresmes and Charlène Brillard for help with the atomic force microscopy, Christophe Boyaval for help with the scanning electron microscopy and Annie Fattorini for help with the metallization. This work was partly supported by the French RENATECH network.

Supplementary References

1. Bowden, N., Huck, W. T. S., Paul, K. E. & Whitesides, G. M. The controlled formation of ordered, sinusoidal structures by plasma oxidation of an elastomeric polymer. *Appl. Phys. Lett.* **75,** 2557–2559 (1999).
2. Owen, M. J. & Smith, P. J. Plasma treatment of polydimethylsiloxane. *J. Adhes. Sci. Technol.* **8,** 1063–1075 (1994).
3. Bayley, F. A., Liao, J. L., Stavrinou, P. N., Chiche, A. & Cabral, J. T. Wavefront kinetics of plasma oxidation of polydimethylsiloxane: limits for sub-µm wrinkling. *Soft Matter* **10,** 1155 (2014).
4. Hillborg, H. *et al.* Crosslinked polydimethylsiloxane exposed to oxygen plasma studied by neutron reflectometry and other surface specific techniques. *Polymer* **41,** 6851–6863 (2000).
5. Görrn, P. & Wagner, S. Topographies of plasma-hardened surfaces of poly(dimethylsiloxane). *J. Appl. Phys.* **108,** 093522 (2010).
6. Tooley, W. W., Feghhi, S., Han, S. J., Wang, J. & Sniadecki, N. J. Thermal fracture of oxidized polydimethylsiloxane during soft lithography of nanopost arrays. *J. Micromechanics Microengineering* **21,** 054013 (2011).
7. Béfahy, S. *et al.* Thickness and Elastic Modulus of Plasma Treated PDMS Silica-like Surface Layer. *Langmuir* **26,** 3372–3375 (2010).

8. Debuisson, D., Dufour, R., Senez, V. & Arscott, S. Wetting on smooth micropatterned defects. *Appl. Phys. Lett.* **99,** 184101–3 (2011).

9. Debuisson, D., Senez, V. & Arscott, S. Tunable contact angle hysteresis by micropatterning surfaces. *Appl. Phys. Lett.* **98,** 184101–3 (2011).

10. Chua, D. B. H., Ng, H. T. & Li, S. F. Y. Spontaneous formation of complex and ordered structures on oxygen-plasma-treated elastomeric polydimethylsiloxane. *Appl. Phys. Lett.* **76,** 721 (2000).

11. Renault, P. O., Villain, P., Coupeau, C., Goudeau, P. & Badawi, K. F. Damage mode tensile testing of thin gold films on polyimide substrates by X-ray diffraction and atomic force microscopy. *Thin Solid Films* **424,** 267–273 (2003).

12. Petersen, K. E. & Guarnieri, C. R. Young's modulus measurements of thin films using micromechanics. *J. Appl. Phys.* **50,** 6761–6766 (1979).

13. Whiting, R. & Angadi, M. A. Young's modulus of thin films using a simplified vibrating reed method. *Meas. Sci. Technol.* **1,** 662 (1990).

14. Gale, W. F. & Totemeier, T. C. *Smithells Metals Reference Book*. (Butterworth-Heinemann, 2003).

15. Cordill, M. J., Taylor, A., Schalko, J. & Dehm, G. Fracture and Delamination of Chromium Thin Films on Polymer Substrates. *Metall. Mater. Trans. A* **41,** 870–875 (2009).

16. Jin, H., Lu, W.-Y., Cordill, M. J. & Schmidegg, K. In situ Study of Cracking and Buckling of Chromium Films on PET Substrates. *Exp. Mech.* **51,** 219–227 (2010).

17. Toth, F., Rammerstorfer, F. G., Cordill, M. J. & Fischer, F. D. Detailed modelling of delamination buckling of thin films under global tension. *Acta Mater.* **61,** 2425–2433 (2013).

18. Lourens, J. A. J., Arajs, S., Helbig, H. F., Mehanna, E.-S. A. & Cheriet, L. Critical behavior of the electrical resistance of very thin Cr films. *Phys. Rev. B* **37,** 5423–5425 (1988).

19. Walther, M. *et al.* Terahertz conductivity of thin gold films at the metal-insulator percolation transition. *Phys. Rev. B* **76,** 125408 (2007).

20. Kaiser, N. Review of the Fundamentals of Thin-Film Growth. *Appl. Opt.* **41,** 3053 (2002).
